# Purification of an Inducible DNase from a Thermophilic Fungus

**DOI:** 10.3390/ijms15011300

**Published:** 2014-01-20

**Authors:** Kyle S. Landry, Andrea Vu, Robert E. Levin

**Affiliations:** 1 Department of Food Science, Massachusetts Agricultural Experiment Station, University of Massachusetts, Amherst, MA 01003, USA; E-Mail: kslandry@foodsci.umass.edu; 2 The Stockbridge School of Agriculture, University of Massachusetts, Amherst, MA 01003, USA; E-Mail: avu@cns.umass.edu

**Keywords:** affinity purification, DNase purification, chromatography, thermophilic fungi, affinity membrane purification

## Abstract

The ability to induce an extracellular DNase from a novel thermophilic fungus was studied and the DNAse purified using both traditional and innovative purification techniques. The isolate produced sterile hyphae under all attempted growing conditions, with an average diameter of 2 μm and was found to have an optimal temperature of 45 °C and a maximum of 65 °C. Sequencing of the internal transcribed region resulted in a 91% match with *Chaetomium* sp., suggesting a new species, but further clarification on this point is needed. The optimal temperature for DNase production was found to be 55 °C and was induced by the presence of DNA and/or deoxyribose. Static growth of the organism resulted in significantly higher DNase production than agitated growth. The DNase was purified 145-fold using a novel affinity membrane purification system with 25% of the initial enzyme activity remaining. Electrophoresis of the purified enzyme resulted in a single protein band, indicating DNase homogeneity.

## 1. Introduction

Thermophilic fungi were first officially defined by Cooney and Emerson [1] as fungi having a maximum temperature at or above 50 °C and a minimum of 20 °C for growth. Thermophilic fungi constitute a small group of eukarya that exhibit the ability to thrive at temperatures normally detrimental to the majority of fungal species. Among the roughly 100,000 recorded fungal species, roughly 50 have been described that have the ability to grow successfully at these elevated temperatures [2] with probably more yet to be described.

The ultimate utility of thermophilic fungi lies in their secretory enzymes. These enzymes have the potential to exhibit desirable properties such as high temperature optima and stability [3]. Enzymes from thermophilic fungi have been studied for their suitability in bioprocesses and other industrial applications [4].

Thermophilic fungi are known to secrete a variety of enzymes such as proteases, lipases, and cellulases. Enzymes with obvious potential applications to industry have been studied and characterized from a variety of thermophilic fungi such as *Mucor pusillus*, *Penicillium duponti*, and *Humicola lanuginose* [4]. Examination of extracellular nuclease production by thermophilic fungi is sparse throughout the literature. One of the first attempts to examine the production of extracellular nucleases was by Adams and Deploey [5]. They tested 10 thermophilic fungi and found that all produced an extracellular DNase and that 6 produced and extracellular RNase. Since then, there has been very little effort to characterize extracellular nucleases produced by thermophilic fungi.

## 2. Results and Discussion

### 2.1. Isolation of the Thermophilic Fungus

Seven fungal isolates were collected and tested for DNase activity. Each isolate was plated on yeast protein soluble starch DNase test agar (DYpSs) and incubated at 45 °C and 55 °C for seven days. Three of the initial seven isolates demonstrated DNase activity, which was indicated by a zone of clearing under and around the growth. However, the zones of clearing for these isolates were very weak. To encourage growth of DNase-producing fungi, a DNA enrichment broth (0.25% Salmon sperm DNA, USB Cat# 14405 100 GM; Santa Clara, CA, USA; 10 ppm polymyxin B, 10 ppm penicillin-G, 10 ppm Ampicillin, 10 ppm streptomycin, 0.005% methyl green) was inoculated with pieces of compost and incubated at 55 °C on a shaker set to 115 RPM. The flask was checked every day for signs of de-coloration, and after five days the blue/green color was gone. The liquid culture was plated on YpSs and incubated at 45 and 55 °C. Five isolates were collected and transferred to DYpSs plates and incubated again at 45 and 55 °C for 5 days. Two of the five isolates were positive for DNase activity, one at 45 °C and another at 55 °C. The organism with the highest amount of DNase activity at 55 °C was named TM-417 and used for this study.

**Figure 1.**
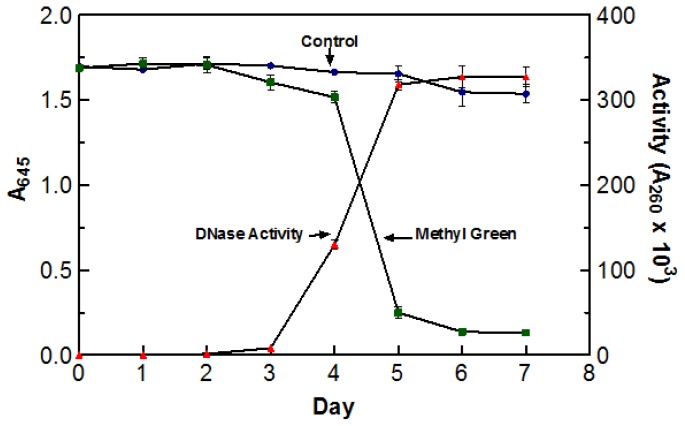
The effect of temperature and DNase activity on the decoloration of the DYpSs liquid media. Based on the results from the control flask, incubation at 55 °C did not have any significant effects on the color intensity of the media. A direct relationship between an increase in DNase activity and the decrease in A_645_ can be seen. Color intensity was measured at 645 nm, which is the optimal absorption wavelength of methyl green. Enzyme activity was measured using the acid soluble assay with a 60 min digestion time at 55 °C. All assays were performed in triplicate.

Due to the high incubation temperatures, it was necessary to make sure that the de-coloration was a direct result of DNase activity and not due to the oxidation of the dye itself. To test this, two flasks containing DYpSs broth were incubated at 55 °C for five days. One flask was inoculated with 5 mL of blended culture and the other was not. The stability of methyl green was measured by recording the absorbance of each flask at 645 nm (abs. max for methyl green). The enzyme activity of the inoculated flask was also tested every day using the acid soluble assay with a 60 min digestion time at 55 °C. There was no significant decoloration in the control sample indicating that the incubation temperature was not responsible for the change in color. The graph clearly depicts the correlation between an increase in DNase activity and a decrease in A_645_ readings. Based on the information presented in Figure 1, optimal DNase activity was achieved after five days (three days agitated and two days static) of incubation at 55 °C. As a result of these findings, crude enzyme sample was harvested after incubating (three days agitated and two days static) at 55 °C for five days.

Initial attempts to identify TM-417 were based on observed morphology using conventional microscopy. The observed hyphae were septated with an average diameter of 2–3 μm. No clamp connections were present on the hyphae; however, there were swollen cells. No fruiting bodies or spores were seen, suggesting that the organism was either in its imperfect stage or was lacking in sexual structures.

**Figure 2.**
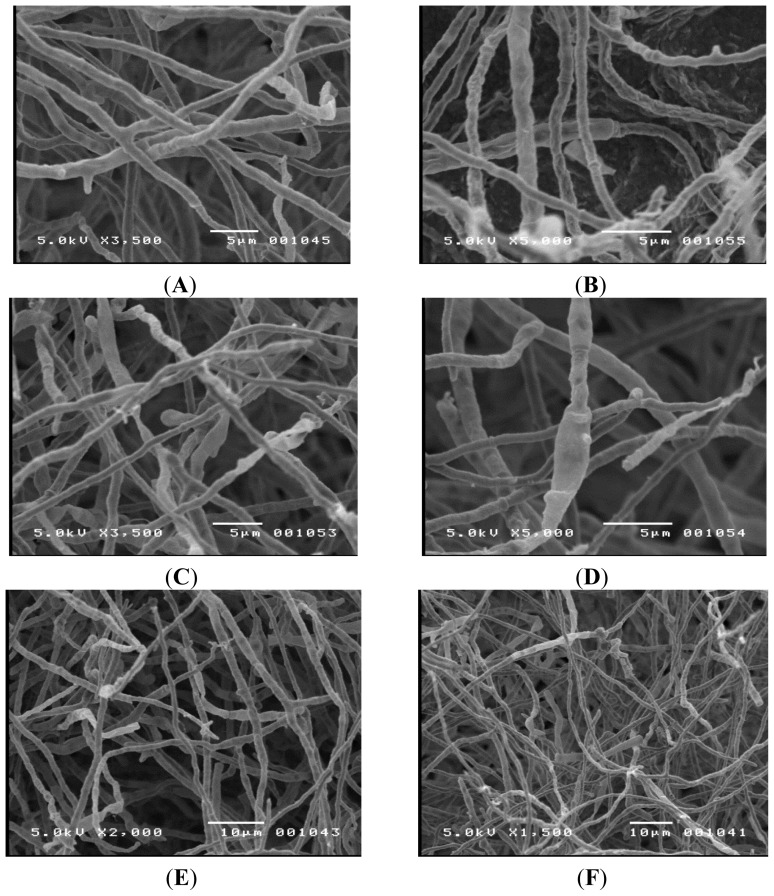
Scanning electron microscopy images of TM-417 performed on a JEOL JSM-5400 scanning electron microscope (JEOL; Tokyo, Japan). No identifying structures were noticed. (**A**) 3500× magnification; (**B**) 5000× magnification; (**C**) 3500× magnification; (**D**) 5000× magnification; (**E**) 2000× magnification; (**F**) 1500× magnification. A swollen cell can be seen in (**D**).

TM-417 was grown on different media (*i.e*., oatmeal, water, yeast glucose, cellulose, and Czapek’s agar), at different pH values, incubated for different time periods, and grown under different lighting conditions to encourage spore formation. All attempts failed. Due to the size of the hyphae, it was difficult to examine the organism. Therefore, scanning electron microscopy was used to get a more detailed picture and the results can be seen in Figure 2. Again, no significant fruiting bodies or spores were seen. Since no identifying structures were observed, the highly conserved ITS region was amplified using the polymerase chain reaction and sequenced using an automated Sanger method. The resulting sequence (Sequence ID # gb|KC462166.1|), was compared to a reference databank using the NCBI Blast program (NCBI; Bethesda, MD, USA) [6]. The organism was identified with a 91% match as a *Chaetomium* sp. indicating that the ITS region of this fungal species was not represented in the GenBank database [7], as it is far below the general acceptance threshold of 98%–100% max identity for species designation.

### 2.2. DNase Production

To generate a sample with a substantial amount of extracellular DNase activity, the isolate TM-417 was grown in a 2 L Fernbach flask with 500 mL YpSs liquid broth containing both DNA and methyl green. The broth was inoculated with 5 mL of a liquid culture that was pulsed five times using a blender. Once inoculated, the culture was placed on a shaker set at 115 RPM and incubated until the blue/green color was gone. With this method, the color in the flask was gone after five days. The optimal temperature for DNase production was found to be 55 °C.

With the intent to decrease the incubation period and increase DNase production, another flask was inoculated and incubated for 3 days at 55 °C on a shaker set to 115 RPM. After the 3 days, the shaker was stopped and the culture was allowed to incubate statically until the coloration was gone. Fungi grown in a static liquid broth develop into a dense fungal mat, whereas agitated fungi develop into individual spheres. It is known that fungi grown statically results in higher amounts of enzyme activity than the activity achieved with agitated growth. With static growth, coloration in the flask was gone after an additional two days of incubation. The overall incubation time did not decrease with static growth; cultures grown in the presence of DNA demonstrated a 269% increase in enzyme activity compared to cultures grown with agitation (data not shown).

### 2.3. Induction Study

**Figure 3.**
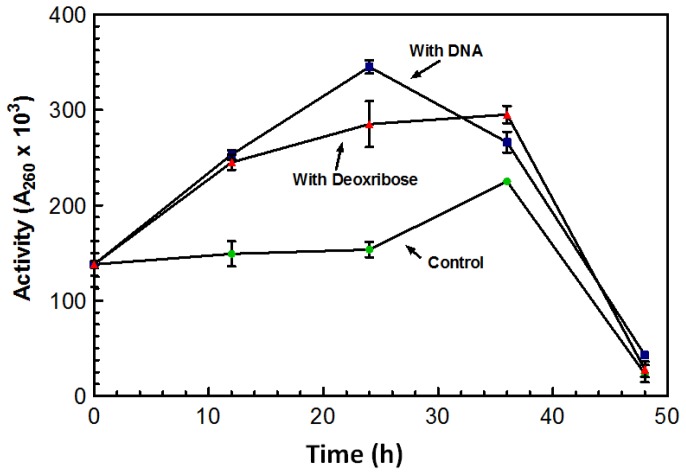
Profile of DNase production in the presence of DNA or deoxyribose. All flasks were incubated for five days (120 h) in the absence of exogenous DNA with agitation (100 RPM). After the fifth day, 1 g of filter sterilized DNA (10 mL) or 1 g deoxyribose was added to a set of flasks and incubated with agitation for 50 h at 55 °C. Control flasks were similarly incubated without the addition of DNA. Activity was measured using the acid soluble assay with a 60 min. incubation time at 55 °C. All assays were performed in triplicate using triplicate flasks.

After growing for five days in the absence of DNA, the overall amount of DNase produced by the culture was significantly lower than cultures grown in the presence of DNA. Flasks in which DNA was added after the five day incubation period showed a spike in DNase activity overnight. Figure 3 represents a typical increase in DNase activity observed after 1 g of DNA was added with activity assessed every 12 h. The sugar 2-deoxy-D-ribose, also known as deoxyribose was investigated as a possible inducing factor for expression of the DNase. Deoxyribose is only found in DNA and makes up the structural backbone of the molecule. The deoxyribose did induce an increase in DNase activity; however, it was not as dramatic as the stimulation by DNA (Figure 3).

Maximum DNase activity was achieved on the third day following the addition of the sugar (Figure 3). With the addition of DNA, the DNase activity reached its maximum after 24 h; activity on the third day was lower than the activity initiated from the deoxyribose (Figure 3). On the third day activity from both the DNA and deoxyribose was almost comparable to that of the control. This decrease in activity may be the result of protease activity that may have leaked out of the aging cells and acting on the nucleases. DNase activity was induced by the presence of deoxyribose suggesting that deoxyribose may play a role in the induction of the DNase.

### 2.4. Effect of Temperature on DNase Production

**Figure 4.**
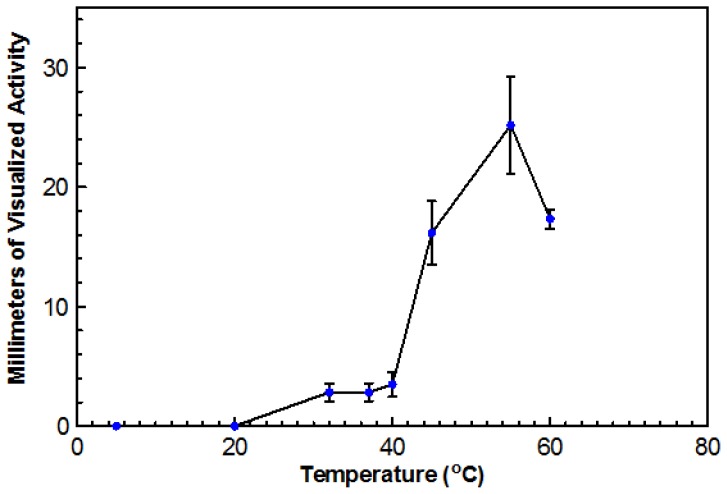
The effect of temperature on DNase production by TM-417. DYpSs agar plates were inoculated with a 10 mm plug taken from a stock culture plate. The plates were incubated at various temperatures (5, 32, 37, 45, 55, and 60 °C) for five days. The zones of clearing were measured in four different directions from the edge of visible growth to the furthest spot of clearing. The mean values and standard deviations from triplicate assays were plotted.

**Figure 5.**
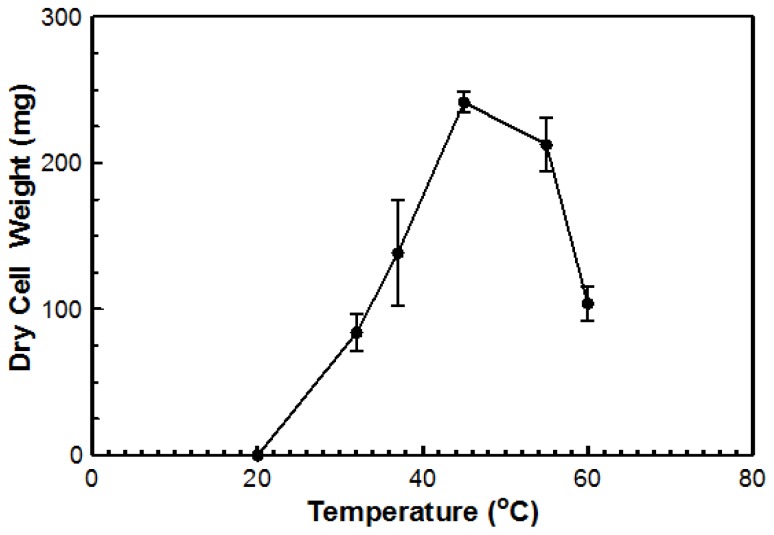
Dry cell mass of fungal growth in a liquid culture at various temperatures. Samples were filtered, rinsed (0.1 M ammonium acetate) and dried at 55 °C overnight prior to weighing. All temperatures were assessed in triplicate.

Using DYpSs, activity was detected from 32 to 60 °C with the highest amount of activity at 55 °C (Figure 4). Based on dry cell mass, the optimal temperature was again determined to be 45 °C with an overall dry cell weight of 241 g (Figure 5). No growth occurred at 20 °C for either method. This organism can be considered a true thermophile since the definition, as provided by Cooney and Emerson [1], states that a fungal thermophile must not grow at or below 20 °C and have a maximum temperature at or above 50 °C.

### 2.5. Purification of the Fungal Thermophilic DNase

Purification procedures are summarized in Table 1. Following filtration of the culture, the enzyme activity of the crude sample was analyzed for protein content and initial DNase activity using the acid soluble assay at 55 °C. Crude sample (250 mL) was dialyzed for 12 h in deionized water containing 0.02% sodium azide and then concentrated to 39 mL using ultrafiltration (10 kDa cutoff) at room temperature, resulting in a concentration factor of 6.41. Ultrafiltration not only concentrated the sample, but also removed any extraneous protein under 10 kDa, explaining the decrease in protein content observed in the concentration step. Initially the crude sample had 320 mg of protein. After dialysis and concentration, the overall protein content was reduced to 35 mg (Table 1).

The enzyme activity of the concentrated sample was assayed for DNase activity. Results from both the crude and concentrate assays were plotted and their slopes analyzed. If no enzyme activity was lost during the concentration, then the rate or slope of the concentrated sample, when divided by the concentration factor, would equal that of the crude.

**Figure 6.**
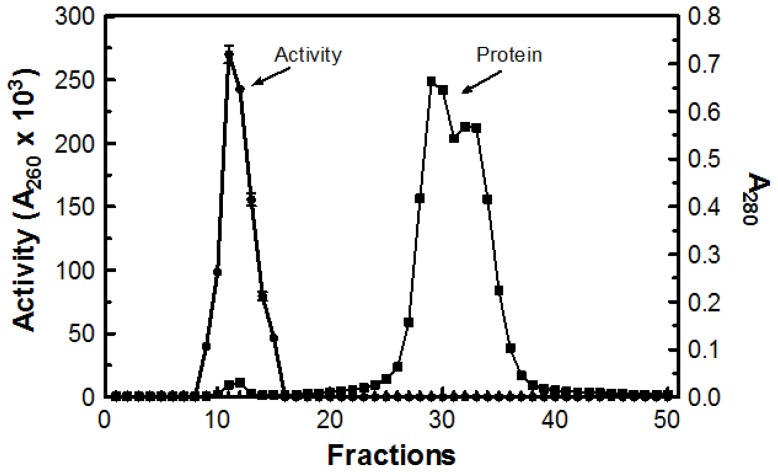
Purification profile of DNase activity and protein. Concentrated sample (1 mL) was passed through a Sephadex G-50 column at 2–5 °C. The DNase activity was measured using the acid soluble assay at 55 °C and protein was estimated by determining the absorbance at 280 nm.

When the slope of the concentrated sample (6.3 × 10^−3^) was divided by the concentration factor (6.41), the resulting slope was 98% of the original crude slope (1.0 × 10^−3^), indicating that only 2% of the total enzyme activity was lost during the concentration process. Overall, there was an 8.96-fold increase in specific activity when compared to the original crude sample (Table 1). Next, 1.0 mL of concentrated sample was passed through a Sephadex G-50 (MP Biomedicals Cat# ICN19558010; Solon, OH, USA) column. The void volume for the column was determined to be 52.8 mL using Blue Dextran 2000 (GE Healthcare Cat# 45-000-048; Buckinghamshire, UK). The first fraction demonstrating a measureable absorbance reading at 280 nm was fraction 9 with additional readings extending to fraction 50. Only fractions 9 through 15 demonstrated DNase activity (Figure 6). Tubes with DNase activity were pooled together for a total volume of 46 mL and the remaining fractions demonstrated no activity.

Originally, final purification was attempted using ceramic hydroxyapatite chromatography. This yielded insufficient enzyme activity for any characterization studies (Data not shown). As a result of this inadequate enzyme recovery, a novel affinity membrane purification system was used as an alternative.

**Figure 7.**
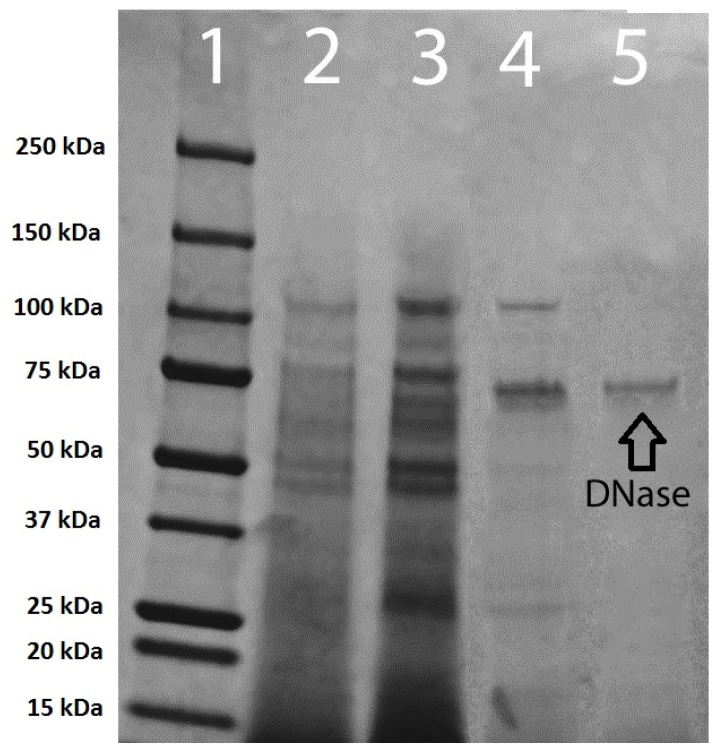
Electrophoresis of the various stages of DNase purification on a 4%–15% gradient polyacrylamide gel. (**1**) 250–10 kDa protein ladder; (**2**) crude DNase; (**3**) concentrated DNase; (**4**) Sephadex G-50 purified DNase; and (**5**) membrane purification following Sephadex G-50 column purification.

Four pooled Sephadex G-50 refrigerated column runs (total volume of 184 mL) were concentrated to 45 mL using the pressurized ultrafiltration cell as described earlier. From this, 10 mL the DNase was purified using 10 DNA coated polyvinylidene fluoride (PVDF) membranes. All 10 membranes were eluted, one at a time, in the same 10 mL of elution buffer. This process was performed for the remaining 35 mL as well, resulting in the utilization of 45 membranes and a final sample volume of 45 mL. Following the elution process, the final sample was dialyzed for 12 h in deionized water containing 0.02% sodium azide at 5 °C and assessed for DNase activity and purity. Using this method, 25% of the DNase was recovered resulting in a 145-fold increase in specific activity when compared to the crude sample (Table 1). This purification procedure resulted in a final reduction in protein by at least 61.4-fold starting with the ultrafiltration concentrate, with a final protein content of 0.57 mg (Table 1). Polyacrylamide gel electrophoresis of pooled membrane purification samples concentrated from 40 to 15 mL indicated the presence of only one protein band (Figure 7).

## 3. Materials and Methods

### 3.1. Isolation of a DNase Producing Thermophilic Fungus

Samples of compost were taken from the University of Massachusetts, Amherst and various farms from the Amherst, MA, USA area and incubated for three days at 55 °C. Small amounts of incubated compost were spread onto yeast protein soluble starch agar plates (YpSs; 0.4% yeast extract, 0.1% K_2_HPO_4_, 0.05% MgSO_4_, 1.5% soluble starch, pH 7.3) and allowed to incubate at 45 and 55 °C. To prevent bacterial growth, an antibacterial cocktail was added to the YpSs plates which included 10 ppm of each of the following: polymyxin B, penicillin G, ampicillin, and streptomycin all of which were filter sterilized. For five days following the initial inoculation, the plates were examined for fungal growth under a dissecting microscope. Any visible growth was transferred to YpSs plates and incubated at 45 and 55 °C until substantial visible growth was observed.

### 3.2. Sequencing

Genomic DNA was extracted with the DNeasy Plant Mini kit (Qiagen, Valencia, CA, USA) from cultures grown on YpSs media at 45 °C for five days. Mycelium was scraped from solid media with a sterile scalpel blade. DNA from the internal transcribed spacer (ITS) region was amplified with PCR, using primers ITS4 and ITS5 [8]. The resultant 529 bp fragment was sequenced and searched against the GenBank database with BLASTn (NCBI; Bethesda, MD, USA) [6].

### 3.3. Initial DNase Detection

The isolated culture was plated on a yeast protein soluble starch medium [1] that was modified to test for DNase activity (DYpSs; 0.4% yeast extract, 0.1% K_2_HPO_4_, 0.05% MgSO_4_, 1.5% soluble starch, 0.005% methyl green, 0.2% DNA (Salmon sperm DNA, USB Cat# 14405 100 GM, Santa Clara, CA, USA; 1.5% agar, adjusted to pH 7.3). The plates were inoculated and incubated in a covered container with moistened paper towels for seven days at 45 and 55 °C. Following the incubation period, the plates were examined for a zone of clearing which indicated DNase activity.

### 3.4. Preparation of Sample for DNase Purification

Isolated cultures were transferred to Fernbach flasks containing 500 mL of DYpSs broth. Each flask was placed on a shaker set to 115 RPM and incubated at 55 °C. After three days the flasks were allowed to incubate statically for an additional 4 days. Cell mass was removed from the sample by vacuum filtration through coarse filter paper (Fisherbrand Filter Paper P8; Pittsburgh, PA, USA). The filtrate was filtered under vacuum through medium (Fisherbrand Filter Paper P5; Pittsburgh, PA, USA), and then through fine filter paper (Fisherbrand Filter Paper P2; Pittsburgh, PA, USA). The filtered sample represented the crude enzyme. To inhibit any bacterial and/or fungal growth, 0.02% sodium azide was added to the crude enzyme sample. All crude enzyme samples were stored at ambient temperature.

### 3.5. Scanning Electron Microscopy

An aliquot of a liquid culture was placed into 20× the volume of fixative (2.5% glutaraldehyde in 50mM sodium phosphate buffer, pH 7.2) for 2 h, rinsed with buffer (50 mM sodium phosphate buffer, pH 7.2) and then secondarily fixed with buffered 1% osmium tetroxide for 1 h. The sample was washed with distilled water, dehydrated with an ethanol series (30%, 50%, 70%, 95%, 100%), loaded into micro-porous specimen capsules (emsdiasum.com; #70187-10; Hatfield, PA, USA) in 100% ethanol and critical point dried in a Balzers CPD-030 (Bal-Tec Inc; Blazers, Liechtenstein) dryer.

Dried samples were placed onto contact adhesive on aluminum SEM stubs, and the surface of the stub up to and just contacting the sample was painted with graphite paint (Ted Pella, #16053; Redding, CA, USA). Samples were sputter coated with approximately 16 nm of gold-palladium (2.2 kV, 5 mA, 3 min) in a Polaron E-5100 sputter (Quorum Technologies; East Sussex, UK) operated with argon backfill. Samples were observed at 5 kV accelerating voltage in a JEOL JSM-5400 SEM. Images were taken from the video capture output with a Scion AG-3 Frame grabber (Scion Corp., Frederick, MD, USA) and NIH Image version 1.60 (NIH; Bethesda, MD, USA) running on a Macintosh 8100 (Apple Inc; Cupertino, CA, USA). Scanning electron microscopy was performed on a JEOL JSM-5400 scanning electron microscope (JEOL; Tokyo, Japan).

### 3.6. Optimal Growth Temperature

The optimal growth temperature of the isolated fungus was assessed two ways: (1) measurable growth on agar plates; and (2) dry cell mass after growth in liquid culture. For the first method, YpSs agar plates were inoculated with a 10 mm plug of the isolate taken from a stock culture. The plates were partially sealed with parafilm and incubated at various temperatures (20, 32, 37, 45, 55, and 60 °C) for five days. The diameter of the fungal growth was measured in four different directions and the average size was determined. For the liquid culture method, a culture was grown in YpSs broth for 5 days at 45 °C on a shaker set to 115 RPM. The culture was loaded into a sterile blender bottle and pulsed five times using an Oster Osterizer Classic^©^ blender (Oster; Boca Raton, FL, USA). Blended culture (1 mL) was transferred to a sterilized 250 mL baffled flask containing 100 mL of YpSs broth. Inoculated flasks were incubated at various temperatures (20, 32, 37, 45, 55, and 60 °C) on a rotary shaker set to 115 RPM for five days. Following the incubation period, a Buchner funnel lined with medium filter paper was used to filter out mycelia. The mycelia were washed with 250 mL of 0.1 M ammonium acetate to remove any trace nutrients. The mycelial mass was dried at 55 °C overnight and weighed the next day.

### 3.7. DNase Induction Studies

Blended culture (1.0 mL) was transferred to a sterilized 250 mL baffled flask containing 100 mL of YpSs broth. The culture was allowed to incubate for 5 days at 55 °C on a shaker set to 100 RPM. After the incubation period, DNase activity was measured using the acid soluble assay with a digestion time of 60 min at 55 °C. 1.0 g of DNA (Fish sperm DNA, USB Cat# 14405 100 GM; Santa Clara, CA, USA) or 2-deoxy-D-ribose (Acros Chem, Cat # AC15328-0050; Geel, Belgium) was dissolved in 10 mL of deionized water, filter sterilized, and added to the 5 day old flasks. The flasks were incubated for another 4 days under the same conditions. DNase activity was measured every day following the addition of the sterilized DNA (USB Cat# 14405 100 GM; Santa Clara, CA, USA)/2-deoxy-D-ribose ribose (Acros Chem, Cat # AC15328-0050; Geel, Belgium). To analyze the enzyme activity in each flask, 4 mL was removed and centrifuged at 13,400 RPM for 10 min. The resulting supernatant was assayed for enzyme activity using the acid soluble assay with a 60 min digestion time at 55 °C.

### 3.8. Effect of Temperature on DNase production

A correlation between temperature and DNase production was established by inoculating the modified yeast protein soluble starch agar plates with a 10 mm plug taken from a stock culture plate. The plates were incubated at various temperatures (2, 20, 32, 37, 45, 55, and 60 °C) for five days. The zones of clearing were measured in four different directions from the edge of visible growth to the furthest spot of clearing.

### 3.9. Membrane Preparation

Membranes were prepared as described by Landry and Levin [9]. A 47 mm, 0.2 μm FP-Vericel membrane (Pall Life Sciences Cat# 66477; Radnor, PA, USA) was rinsed through a Millipore^©^ (Billerica, MA, USA) membrane filtration unit, under vacuum with 200 mL of deionized water. To produce single stranded DNA, 1.0 g of DNA (Fish salmon sperm, USB Cat# 14405 100 GM; Santa Clara, CA, USA) was dissolved in 50 mL of 2× SSC buffer (1.75% sodium chloride, 0.88% sodium citrate, adjusted to pH 7.0) and boiled for 1 min in a 200 mL beaker. The beaker was then placed in ice and cooled to 10 °C. Once at the desired temperature (~10 °C) the rinsed membrane was added to the beaker and placed in an iced bucket and subjected to rotary agitation (75 RPM) for 60 min. After 60 min the membrane was removed, rinsed with deionized water and dried using a stream of hot air.

### 3.10. Sample Purity

Electrophoresis was carried out in a vertical Bio-Rad Mini-Protean^®^ system (Hercules, CA, USA) using a Tris/Glycine/SDS buffer (Bio-Rad Cat# 161-0772; Hercules, CA, USA). The sample was diluted 1:1 with a Laemmli buffer solution containing 950 μL Laemmli buffer and 50 μL β-mercaptoethanol (Laemmli buffer Bio-Rad Cat# 161-0737; Hercules, CA, USA)/β-mercaptoethanol (Fisher Scientific Lot# 940723; Pittsburgh, PA, USA). The mixture was heated for 5 min at 99 °C in a thermal cycler (Techne Model FTGENE5D; Minneapolis, MN, USA). Proteins were separated using 4%–15% gradient Mini-Protean^®^ TGX^™^ (Bio-Rad Cat# 456-1083; Hercules, CA, USA) 30 μL well precast gels. Gels were loaded with 30 μL of sample and run at 155 V for 45 min. Gels were placed on a shaker set to low speed (25 RPM) and stained using 50 mL of Acqua Stain (Bulldog Bio Co. Cat# AS001000; Portsmouth, NH, USA) for 60 min. The bands were photographed with a PowerShot G10 Digital Canon Camera (Tokyo, Japan) equipped with an orange filter lens. A 250–10 kDa protein ladder (Dual Color Precision Plus Protein Standards. Bio-Rad Cat#161-0374; Hercules, CA, USA) was used as a standard.

### 3.11. Determination of Protein Content

Protein was determined by the Lowry method [10] or by measuring absorbance at 280 nm. A blank was generated by substituting the sample with deionized water.

### 3.12. Acid Soluble Assay for Enzyme Activity

DNase activity was determined by measuring acid soluble nucleic acids. The method used in this study was a modified version of Eaves and Jeffries [11]. Enzyme sample (0.75 mL) was added to 0.75 mL of substrate (Fish sperm DNA, USB Cat# 14405 100 GM; Santa Clara, CA, USA; 10 μmol MgSO_4_ in 0.1 M imidazole buffer, pH 7.0) and incubated in a controlled water bath at 55 °C for 10 min. The reaction was stopped by adding 0.5 mL of uranylacetate-perchloric acid reagent (0.25% uranylacetate in 10% perchloric acid). Reaction tubes were cooled in an ice bath for 15 min. The mixture was diluted with 2.0 mL of deionized water and the precipitate removed by centrifugation at 13,400 RPM for 5 min at ambient temperature. The absorption at 260 nm was measured against a reagent blank prepared by adding the uranylacetate-perchloric acid reagent to the substrate prior to the addition of the enzyme. One unit of enzyme activity is defined as an increase in absorbance of 0.05 units in a cuvette of 1 cm light path at 260 nm.

### 3.13. Ultrafiltration Membrane Concentration

Crude enzyme sample (250 mL) was dialyzed against deionized water (containing 0.02% sodium azide) for 12 h using dialysis tubing (Fisherbrand #21-152-5 Cat# 21-152-5; Pittsburgh, PA, USA) having a flat width of 40 mm and a molecular weight cut-off of 6–8 kDa. The dialyzed crude enzyme sample was then concentrated using a pressure cell (Amicon^©^; Cat# 5124; Billerica, Ma, USA; 500 mL pressure cell) and a 10 kDa regenerated cellulose membrane (Millipore^©^ Utrafiltration YM10 Dia. 76 mm, Cat# 13642; Billerica, MA, USA). The resulting sample was designated the concentrated enzyme sample.

Prior to sample filtration, 200 mL of distilled water followed by 100mL of 1% (*w*/*v*) bovine serum albumin (Fisher Biotech, Fraction V, Lot BP1605-1; Pittsburgh, PA, USA) solution was passed through the pressure cell/membrane apparatus. This was to ensure that no protein from the sample would bind to the membrane.

### 3.14. Sephadex G-50 Column Chromatography

Sephadex G-50 (MP Biomedicals Cat# ICN19558010; Solon, OH, USA) was hydrated in deionized water for 3 h at 100 °C prior to loading the column. A 45 cm × 2.5cm glass column was used. A 34 cm long column of Sephadex G-50 was equilibrated for 24 h with 0.5 M imidazole buffer (pH 7.0) containing 0.02% sodium azide. A portion (1 mL) of concentrated sample was loaded onto the bottom of the gel bed and eluted ascendingly (0.5 M imidazole buffer (pH 7.0) containing 0.02% sodium azide) at a rate of 2 mL/min (120 mL/h) in a 2–5 °C chromatography refrigerator. Fractions (6.6 mL) were collected using a Gilson FC 203B fraction collector (Middleton, WI, USA). All fractions which demonstrated DNase activity were pooled together. Activity was measured using the acid soluble assay at 55 °C with a digestion time of 20 min.

### 3.15. Affinity Membrane Purification

To purify the DNase, 10 mL of sample from four concentrated Sephadex G-50 column runs (45 mL total volume) was chilled to 5 °C in a 200 mL beaker and placed in an iced bucket on a shaker set to 50 RPM in a 2 °C refrigerator. A DNA coated membrane (preparation previously described in Section 3.9) was added to the chilled sample and removed after three min. The membrane was removed, rinsed quickly with deionized water and transferred to another beaker on ice in a 2 °C refrigerator containing the elution buffer (0.5% NaCl, 0.5 M imidazole, adjusted to pH 7.0). The buffer was previously chilled to 5 °C prior to elution. The remaining 35 mL of concentrated Sephadex G-50 sample was purified in the same manner. All membranes were used once and discarded.

## 4. Conclusions

An extracellular DNase was purified and characterized from an unidentified thermophilic fungus using a combination of traditional and novel purification techniques. The fungus demonstrated an optimal growth temperature of 45 °C which is quite common among thermophilic and thermotolerant fungi. The lack of any spores or sexual structures is to be noted, considering every documented thermophilic ascomycete produces some form of ascospore [12]. It should be noted that temperature does play an influential part in the formation of ascospores in thermophilic ascomycetes. For example, *Chaetomium thermophile* was found to produce ascospores at 45 °C but at higher temperatures only produced sterile ascocarps or mycelium [13]. It is possible that TM-417 may also have temperature requirements for ascospore development, however, all of the various media and environmental conditions used failed to stimulate spore production, suggesting that this is either a sterile fungus or one that requires extremely specific, and yet to be determined conditions to enter its sexual stage.

The purification of the DNase was achieved using size exclusion chromatography and an affinity based membrane purification system. This combination of purification techniques led to a 145-fold increase in specific activity, with 25% of the initial enzyme activity. The final purified enzyme sample was tested for RNase activity to see if the purified enzyme was a non-specific nuclease; no RNase activity was detected. The method used to detect RNase activity was identical to the described acid soluble assay method except yeast RNA (yeast RNA; 1mg/mL (Sigma R6625 Cat# R6625; Milwaukee, WI, USA) was substituted for the salmon sperm DNA.

The membrane based system worked well and was faster than traditional column chromatography. However, this technique was more labor intensive. Efficiency of this technique may be improved if the procedure was optimized further. Conversely, this method resulted in the visualization of a single protein band suggesting enzyme homogeneity, and demonstrating the effectiveness of this technique. Since separation was achieved with minimal loss of activity, this technique could be utilized during the final steps of purification for other DNA specific enzymes where loss of a high level of activity is not acceptable.

The final degree of sample purity was acceptable for the characterization of the DNase. The thermal stability of the DNase was found to be significant, yet not necessarily novel. Extracellular thermophilic enzymes have to be robust since they are secreted into an environment often lacking of protective buffer salts or cellular compartments. Nucleases from other thermophilic and thermotolerant fungi also demonstrate similar thermal stability. For example, the extracellular enzyme Nuclease S1, originally isolated from a digestive enzyme preparation from *Aspergilus oryaze*, has thermal stability with strong nucleolytic activity at 65 °C [14,15]. Finally, the novel method of affinity membrane purification described here was successfully applied for purification of the TM-417 thermophilic nuclease, suggesting that this method may be useful for the purification of other DNA binding enzymes from other biological systems.

## Figures and Tables

**Table 1.** Overall purification of the thermophilic DNase a.

Fraction	Vol (mL)	Protein content (mg)	Total activity (U)	Sp act (U/mg)	Recovery (%)	Increase in sp act
Crude	250.0	320.0	1408.5	4.40	100	1
Concentrated	39.0	35.0	1379.26	39.41	97.9	8.96
Sephadex G-50	46.2	2.8	854.98	305.35	60.70	69.40
Membrane	45	0.57	365.12	640.56	25.22	145.58

a One unit of enzyme activity is defined as an increase in absorbance of 0.05 units in a cuvette of 1 cm path length at 260 nm using the acid soluble assay at 55 °C.
